# Smoking Status, Changes in Smoking Status and Health-Related Quality of Life: Findings from the SUN (“Seguimiento Universidad de Navarra”) Cohort

**DOI:** 10.3390/ijerph6010310

**Published:** 2009-01-19

**Authors:** Mario Guitérrez-Bedmar, María Seguí-Gómez, Enrique Gómez-Gracia, Maira Bes-Rastrollo, Miguel A Martínez-González

**Affiliations:** 1 Universidad de Málaga (Spain), Dpt. of Preventive Medicine and Public Health / School of Medicine, Campus de Teatinos, 29071 Málaga, Spain; E-Mails: bedmar@uma.es (M. G.-B.); gomez_gracia@uma.es (E. G.-G.); 2 Universidad de Navarra (Spain), Dpt. of Preventive Medicine and Public Health / School of Medicine, Irunlarrea 1, Edif. Investigación, 31080 Pamplona, Spain; E-Mails: mbes@unav.es (M. B.-S.); mamartinez@unav.es (M. A. M.-G.)

**Keywords:** Health related quality of life, SF-36, Tobacco

## Abstract

We aimed to evaluate the association between smoking, changes in smoking, and quality of life in a cohort of Spanish university graduates. Smoking habits were self-reported at baseline and four years later. Quality of life was assessed using the Short Form-36 (SF-36) at year 4. Statistical differences in SF-36 scores between groups were determined using ANCOVA with age and sex as covariates. Out of 5,234 eligible participants over 2000–2006, there were 2,639 non-smoker participants, 1,419 ex-smokers, and 1,048 smokers. Within the previous four years, 435 participants became recent quitters and 205 starters. Comparing smoking and health status in year 4, non-smokers showed better scores than the other categories of ever smoking in all dimensions except in the vitality scale value, which was similar in non-smokers and in those smoking less than 15 cigarettes/day. Comparing changes in smoking and health in year 4, continuing smokers had statistically significant worse scores than non-smokers in general health, social functioning, role-emotional and mental health, whereas recent quitters showed statistically significant improvements in role-emotional and mental health over those who had continued smoking or those who became smokers. Our findings support a dose-response relationship between cigarette consumption and a worse quality of life in general and mental health in particular. They also support that changes in smoking have an impact on health.

## 1. Introduction

Smoking is a well-known cause of mortality and its adverse effects are described in numerous studies around the world. Tobacco consumption in Spain causes the death of one out of four men and one out of fifty women [1]. Beyond its impact on mortality, smoking also is associated with morbidity and disability [2], although the impact of such conditions is far less known.

From a public health perspective, this is a crucial time to educate the population on the tobacco-related burden of disease and its impact not only in morbidity and mortality but also on health-related quality of life (HRQL). This endeavour may help to show that efforts like the recently implemented legislation are not in vain [3]. We support the view that all the studies about adverse effects of tobacco consumption are welcome in order to join forces for a smoke-free world [4]. We also believe it interesting to investigate whether recent changes in smoking status contribute to changes in HRQL, as a possible incentive to quit smoking. This should be relevant to all public health professionals interested in promoting healthier lifestyles.

Thus, the aim of our study was to evaluate the association between smoking or longitudinal changes in smoking status and HRQL in a dynamic cohort of university graduates. University graduates play a leadership role in societies, and the results obtained in this cohort may be an advance outcome of the general population status in a short- or medium-term.

## 2. Methods

### 2.1. Study Population

The Seguimiento Universidad de Navarra (SUN Project) is a dynamic prospective follow-up (cohort) study of university graduates through Spain. This cohort follows a methodology similar to that used in large American cohorts such as the Nurses’ Health Study and the Health Professionals Follow-up Study. A detailed description of SUN Project can be found in previously published papers [5] or at www.unav.es/departamento/preventiva/SUN.

In short, participant recruitment started in December 1999 and information is collected via paper or web-based self-administrated questionnaires. Participants are invited to submit their follow-up questionnaires on a biennial basis. As of February 2006, the SUN Project had carried out three assessments. The first one (Q0 or baseline) gathers information about socio-demographic variables, lifestyle factors (including smoking), clinical variables and a detailed semi-quantitative food frequency questionnaire. More than 18,000 people have become participants and have completed this baseline assessment. The four-year follow-up questionnaire (Q4) also gathers information about changes in lifestyle (including smoking), food and medication consumption, and the onset of diseases and injuries, and it incorporates the 36-item short form (SF-36) questionnaire. We used the data of the participants who were eligible to submit their four-year follow-up questionnaire as of February 2006. The cohort study has been reviewed and approved by the Ethics Committee of the Universidad de Navarra.

### 2.2. Assessment of HRQL

HRQL was assessed in Q4 with a validated Spanish version of the SF-36 Health Survey [6]. This questionnaire contains 36 items which measure eight multi-item parameters of health status: physical functioning, role limitations due to physical health problems (role-physical), bodily pain, general health perceptions, vitality, social functioning, role limitations due to emotional problems (role-emotional) and mental health. The first four domains deal with physical aspects, and the next four reflect psychological features. For each parameter, scores are coded, summed and transformed to a scale from 0 (the worst possible condition) to 100 (the best possible condition).

### 2.3. Assessment of Smoking

Tobacco consumption was self-reported at Q0 and Q4 using the same question and possible categories. For the cross-sectional analysis on the relationship between smoking and health, we classified subjects using their responses to Q4 into non-smokers, ex-smokers, and smokers. The latter were further subdivided into those smoking less than 15, between 15 and 24 and 25 or more cigarettes/day.

For the analyses on the relationship between changes in smoking in the previous 4 years and health, information on whether changes in smoking status had occurred were derived from the answers to both Q0 and Q4. Participants were classified into non-smokers, current-smokers, recent quitters or starters (although some of these starters may actually be relapsers). Non-smokers included either participants who reported not smoking either in Q0 or in Q4 or those who were ex-smokers in Q0 and remained so in Q4. Current-smokers were those who reported smoking in both Q0 and Q4. Recent quitters were those who reported being smokers in Q0 but reported having quitted smoking between Q0 and Q4. Starters were participants who reported being current smokers in Q4 but had told us they were not smokers in Q0.

### 2.4. Statistical Analyses

Means and 95% confidence intervals are reported for descriptive purposes only. Age and sex adjusted mean scores for each dimension of the SF-36 were estimated using multiple least square regressions for each of the eight dimensions. For each estimated regression equation, we replaced age and sex variables with their respective average (mean) values. Statistical differences in mean scores between groups of smoking status and between changes in smoking status were determined by ANCOVA, using the SPSS General Linear Model procedure with age and sex as covariates. Whether these means were statistically significantly different (defined as a two tailed p value ≤0.05) was evaluated *post hoc* using the Bonferroni correction. We used ANCOVA to allow for the adjustment of possible confounders such as age and gender and to accommodate for the imbalanced nature of the sample size of the groups being compared.

Beyond statistical significance, 5-point differences (in the 0 to 100 scale for each of the eight SF36 dimensions) were considered to be of clinical relevance [7]. That is, participants′ groups whose scoring is 5 points or more apart have clinically identifiable differences in their health status.

## 3. Results

Up to February 2006, 5,234 participants had returned their Q4 questionnaires. Smoking status at Q4 was missing for 128 participants. Therefore, we analysed data from 5,106 participants. According to their answers in Q4, 2,639 (51.7%) were non-smokers, 1,419 (27.8%) were ex-smokers and 1,048 (20.5%) were smokers. Smokers consisted of 730 (69.6%) participants smoking less than 15 cigarettes/day, 242 (23.1%) smoking between 15 and 24 cigarettes/day and 76 (7.3%) smokers of 25 or more cigarettes/day.

Concerning smoking changes between Q0 and Q4, 5,052 (98.9%) participants reported their smoking status in both questionnaires. Among them, 3,594 (71.1%) participants continued to be non-smokers (or had remained as ex-smokers since Q0), 818 (16.2%) continued to be smokers, 435 (8.6%) were recent quitters, and 205 (4.1%) were starters.

The gender and age distribution of participants by smoking status are presented in Table 1. Ex-smokers were older than those smoking less than 15, 15–24 or 25 or over cigarettes/day. The proportion of women was higher among those smoking less than 15 cigarettes/day and non-smokers than among smokers of 25 or more cigarettes/day (67.4 and 63.3% respectively). With respect to smoking-changes, starters were, on average, younger than the other groups and there were no significant differences in their sex distribution.

Table 2 shows the age- and sex- adjusted mean scores of each smoking group on the eight dimensions of the SF-36. Overall, the SF36 scores for all eight dimensions were statistically significant between smoker-status groups (p<0.05).

The non-smoker group showed better SF-36 mean scores than the other groups in all dimensions, with the exception of their vitality scale value being similar to that of participants smoking less than 15 cigarettes/day. Many of these differences reached statistical significance as seen in Table 2. Ex-smokers had significantly lower (i.e., worse) SF-36 mean scores than non-smokers in role-physical and bodily pain. The SF-36 mean scores of those smoking less than 15 cigarettes/day were significantly worse than non-smokers for role-emotional and mental health. Participants smoking less than 15 and those smoking 25 or more cigarettes/day had significantly worse SF-36 mean scores than non-smokers for five dimensions: physical functioning, general health, social functioning, role-emotional and mental health. Differences between smokers of 15–24 cigarettes/day and ex-smokers’ SF-36 mean scores were significant for general health and role-emotional. Ex-smokers had significantly better SF-36 mean scores than those smoking 25 or more cigarettes/day for general health, social functioning, role-emotional and mental health. There were important differences within the smokers’ groups. Participants smoking less than 15 cigarettes/day had significantly better SF-36 mean scores than those smoking 15–24 cigarettes/day for physical functioning, general health and vitality. However, the main differences were between smokers of 25 or more cigarettes/day and those smoking less than 15 cigarettes/day, with the latter group scoring significantly better for six out of eight dimensions.

Also seen in Table 2 is the fact that clinically significant differences are clear between smokers of 25 or more cigarettes/day, non-smokers, ex-smokers and those smoking less than 15 cigarettes/day. These differences are greater for the general health, vitality, social functioning, role-emotional and mental health dimensions. In fact, and for mental health, participants smoking 25 or more cigarettes/day are also clinically worse than those smoking between 15 and 24 cigarettes/day are. Smokers of 25 or more cigarettes/day almost reach the 5-point difference for physical functioning and role-physical when compared to the non-smoker participants.

With regards to changing smoking habits over the 4-year span, Table 3 shows the age and sex adjusted SF-36 mean scores on the eight dimensions of the SF-36. Overall, differences among groups in SF-36 mean scores were statistically significant (p<0.05) for five dimensions: physical functioning, general health, social functioning, role-emotional and mental health.

Current-smokers had worse SF-36 mean scores than non-smokers in all dimensions. These differences were statistically significant for four dimensions: general health, social functioning, role-emotional and mental health. The recent quitters group had significantly better SF-36 mean scores than smokers for general health and role-emotional. Starters reported significantly better SF-36 mean scores than smokers only for general health, and they showed no statistically significant differences with non-smokers or recent quitters.

Clinically significant differences were less common when change in smoking status was evaluated. In fact, only role emotional was clinically lower among current smokers than among non-smokers, recent quitters or starters.

## 4. Discussion

In this cohort of university graduates, we observe a strong association between smoking status and a HRQL both in the cross-sectional comparison of their smoking and health state in the fourth year of their participation in the cohort and in the evaluation of changes in smoking behaviour over the previous four years on their current health state. Generally, smokers showed poorer HRQL than non-smokers. Within smoking groups, SF-36 mean scores were inversely associated with the amount of tobacco smoked. These differences were both clinically and statistically significant.

Interesting differences between smokers and non-smokers were more marked in reference to mental dimensions. This result is in agreement with other studies on different populations. In one of the first studies about smoking and HRQL, Lyons *et al.* investigated quality of life status of “ever” and “never” smokers [8]. Their findings show main differences in three physical dimensions (physical functioning, bodily pain and general health) and only in one mental dimension (vitality). But later studies have expanded on this work. Laaksonen *et al.* [9] showed that middle-age employees of the City of Helsinki who were current smokers had consistently poorer mental health than non-smokers have. Another study showed that never smoker students from two public Brazilian universities reported better mean scores in physical functioning, general health and the four mental dimensions than smokers [10]. A finding again identified in a study of primary care patients by Wolf *et al.* [11] and other studies [12–14], including one on a large representative sample of non-institutionalized Spaniards aged 16 years or older [15]. The nature of this relationship is still unclear and the question of causality is much more complex [16–24]. We look forward to analyzing further this issue as data on changes in smoking status and changes in health related quality of life among SUN cohort participants becomes available.

It is noteworthy to mention that the study by Bellido-Casado *et al.* [25] in a representative sample of the general population over 14 years of age in the western health area of Valladolid (Spain) reported no differences among smokers and non-smokers for either the physical or the emotional dimensions. They argued that Spanish pattern of tobacco consumption is different from those of northern European and Anglo-Saxon countries, but our results seem to suggest that the reason may be more related to their methodology or their sample size instead.

In our results, differences between non-smokers and smokers were more marked for those smoking between 15 and 24 cigarettes/day and especially for smokers of 25 or more cigarettes/day. This dosage-response relationship has been shown in previous studies that compared non-smokers with different levels of tobacco exposure [9, 12, 13]. In fact, Wilson *et al.* [13] encouraged smokers of 25 or more cigarettes/day to become in the group of those smoking less than 15 cigarettes/day in order to improve their quality of life. This may be a first steep in the hard job to total cessation. Our results in the evaluation of whether changes in smoking habits over 4 years relate to health state and those of other studies strengthen this argument [9, 12, 15].

A more important issue in order to promote smoking cessation is the comparison between ex-smokers and smokers. We have not encountered statistically significant differences between ex-smokers and participants smoking less than 15 cigarettes/day; yet, this situation changes when we compare the earlier with those smoking 15–24 cigarettes/day and those smoking 25 or more cigarettes/day There was a dosage-response relationship between cigarette consumption and a worse quality of life in general health and the mental subscales. Other studies reported this association too [9, 12, 13, 15, 25, 26]. Compared with non-smokers, ex-smokers had lower SF-36 mean scores and these differences were statistically significant in role-physical and bodily pain. Wilson *et al*. encountered significantly lower SF-36 mean scores in vitality and the four physical subscales when compared non-smokers with ex-smokers [13]. Mulder *et al*. [12] showed that, compared with ex-smokers, non-smokers had better HRQL in bodily pain only. Other studies had not encountered significant differences between non-smokers and ex-smokers [9, 25].

Bradford-Hill’s criteria for causation may help clarify the effect of smoking in mental health. Our results show consistency, strength of association, a dosage-response relationship and coherence with previous knowledge. Even though temporal sequence cannot be demonstrated with transversal data from Table 2, Table 3 shows a relationship between changes in smoking status and HRQL, especially in mental subscales. Table 3 also shows that short- and medium-term effects of exposure to tobacco seem to correlate particularly with emotional problems.

Concerning short- and medium-term effects of tobacco in HRQL we have found that smoking cessation improves general health and role limitations due to emotional problems. We have not found differences between non-smokers and starters and it may be due to a possibly long-term effect of smoking in HRQL [26]. Mitra *et al.* [27] carried out a similar study on adults with disabilities. They showed that the mean scores of recent quitters were significantly better than smokers mean scores in all dimensions except for physical functioning. We cannot compare our results with those of Mitra *et al.* (2004) because differences between this population and our cohort are too large. Besides Mitra’s study [27], there are no studies that analyze the effect of changes in smoking status over the SF-36 mean scores with this methodology.

### Study limitations and strengths

As with all those other studies cited, our findings rely on the validity of self-reported smoking habit (and changes) and health-related quality of life. Yet, we believe the face validity of these tools has been extensively demonstrated in the literature [28].

Granted our cohort smoking status at baseline (20.5%) is somewhat lower than that of the Spanish general population at a comparable time (28.5%) [29], and its general health status is better than that of the age and gender-adjusted general population (results not shown) [30]. This, we believe to be related with their university graduate status and educational level, has long been known as associated with health status and better knowledge of health-related lifestyles and risk factors [31]. Yet the relationship that we have identified between smoking and health and the modification of smoking habit and quality of life is likely to be generalized to that of the general population.

At the time of the analysis, we used data of the cohort participants who had become eligible for their 4-year follow-up questionnaires. The 5,234 cases available represent some 95% of the eligible patients. Previous evaluations of our dynamic attrition rates confirm its non-biased nature [32].

## 5. Conclusions

Our results suggest that smoking is associated with poorer HRQL especially with mental health and that there is a dosage-response relationship between numbers of cigarettes per day and worse HRQL. Smoking cessation might improve, in the short-term, general health and role limitations due to emotional problems.

## Figures and Tables

**Table 1:** Age (in years) and sex distribution (in % females) by smoking status and changes in smoking status. SUN study participants, N= 5,106.

	Smoking status	N	Age mean (age range 20–87 years) (95% CI)	Sex % female (95% CI)
At 4-year follow-up (Q4)	Non smokers	2,639	34.3 (33.8, 34.7)	63.6 (61.8, 65.5)
	Ex-smokers	1,419	41.0 (40.3, 41.6)	56.7 (54.1, 59.3)
	<15 cigs/day	730	34.1 (33.4, 34.9)	67.4 (63.9, 70.8)
	15–24 cigs/day	242	35.5 (34.2, 36.9)	49.2 (42.8,55.5)
	25+ cigs/day	76	38.7 (36.4, 41.1)	43.4 (32.0, 54.8)
	Total number of smokers	1,732		
	p-value (ANOVA)		<0.001	<0.001

Changes within last 4 years (Q0 and Q4)	Non-smokers	3,594*	36.7 (36.3, 37.1)	61.0 (69.3, 62.5)
	Smokers	818	35.0 (34.3, 35.8)	62.3 (59.0, 65.7)
	Recent quitters	435	35.0 (34.0, 36.1)	64.1 (59.6, 68.7)
	Starters	205	33.3 (31.8, 34.8)	59.5 (52.7, 66.3)
	P-value (ANOVA)		<0.001	0.504

* Comprises the 2,639 subjects who were non-smokers at Q4 and the 995 who reported being ex-smokers since Q0.

**Table 2:** Age and sex adjusted mean scores (95% confidence interval) by smoking status on the SF-36 health status questionnaire. SUN study participants (N=5,106).

SF-36 dimensions	Non smokers (N=2,639)	Ex smokers (N=1,419)	<15 cig/day smokers (N=730)	15–24 cig/day smokers (N=242)	25+ cig/day smokers (N=76)	p-value (ANCOVA)
Physical functioning	95.0 (94.7, 95.4)	94.4 (93.9, 94.9)	94.8 (94.2,95.5)	92.7*+ (91.5,93.8)	91.6*+ (89.5, 93.6)	<0.001
Role-physical	91.5 (90.5, 92.4)	89.0* (87.7, 90.3)	91.5 (89.7,93.3)	91.5 (88.3,94.6)	87.5 (81.9, 93.1)	0.033
Bodily pain	79.8 (79.0,80.6)	77.4* (76.4, 78.5)	79.1 (77.7,80.5)	79.7 (77.2,82.2)	78.2 (73.7, 82.6)	0.013
General health	75.5 (74.9, 76.1)	75.2 (74.3, 76.1)	74.7 (73.5,75.9)	70.5*+# (68.5, 72.5)	68.6*+# (65.0, 72.2)	<0.001
Vitality	66.3 (65.7, 66.9)	65.8 (64.9, 66.6)	66.9 (65.7,68.0)	63.3+ (61.3, 65.4)	61.3+ (57.7, 64.9)	0.002
Social functioning	90.4 (89.7,91.0)	89.0 (88.1, 89.9)	89.0 (87.8,90.3)	86.5* (84.3, 88.6)	83.1*+# (79.2, 86.9)	<0.001
Role-emotional	87.5 (86.3,88.6)	86.6 (85.1,88.2)	82.9* (80.8,85.1)	78.0*# (74.3, 81.8)	71.6*+# (65.0, 78.3)	<0.001
Mental health	76.4 (75.8,76.9)	75.3 (74.6,76.1)	74.7* (73.7,75.7)	73.0* (71.2, 74.8)	68.8*+# (65.6, 72.0)	<0.001

* Statistically significantly lower (p<0.05) than non-smokers. (Bonferroni post-test correction)

+ Statistically significantly lower (p<0.05) than <15 cig/day smokers. (Bonferroni post-test correction)

# Statistically significantly lower (p<0.05) than ex-smokers. (Bonferroni post-test correction)

**Table 3:** Age and sex adjusted mean scores (95% confidence interval) on the SF-36 health status questionnaire according to changes in smoking status during follow-up. SUN study participants (N=5,052).

SF-36 dimensions	Non- smokers (N=3,594)	Smokers (N=818)	Recent quitters (N=435)	Starters (N=205)	p-value (ANCOVA)
Physical functioning	94.9 (94.6,95.2)	94.0 (93.4, 94.6)	94.1 (93.2, 94.9)	94.5 (93.3, 95.8)	0.034
Role-physical	90.9 (90.1, 91.7)	90.5 (88.8, 92.2)	88.7 (86.4, 91.0)	93.2 (89.8, 96.6)	0.163
Bodily pain	79.2 (78.5, 79.8)	78.6 (77.3, 80.0)	77.5 (75.6, 79.3)	81.8 (79.1, 84.6)	0.061
General health	75.4 (74.9 ,75.9)	72.6*+# (71.5, 73.7)	75.8 (74.3, 77.3)	76.1 (73.9, 78.3)	<0.001
Vitality	66.1 (65.6, 66.7)	65.5 (64.4, 66.6)	65.8 (64.3, 67.3)	66.6 (64.5, 68.8)	0.709
Social functioning	90.1 (89.5, 90.7)	87.6* (86.4, 88.7)	88.5 (86.9, 90.1)	90.1 (87.8, 92.5)	0.001
Role-emotional	87.3 (86.4, 88.3)	80.0*+ (78.0, 82.0)	85.9 (83.2, 88.7)	84.9 (80.9, 89.0)	<0.001
Mental health	76.2 (75.7, 76.6)	73.8* (72.9, 74.8)	74.9 (73.6, 76.3)	74.1 (72.2, 76.1)	<0.001

* Statistically significantly lower (p<0.05) than non-smokers. (Bonferroni post-test correction)

+ Statistically significantly lower (p<0.05) than recent quitters. (Bonferroni post-test correction)

# Statistically significantly lower (p<0.05) than starters. (Bonferroni post-test correction)
